# Evaluation of the newly proposed simplified histological classification in Japanese cohorts of myeloperoxidase-anti-neutrophil cytoplasmic antibody-associated glomerulonephritis in comparison with other Asian and European cohorts

**DOI:** 10.1007/s10157-012-0755-7

**Published:** 2012-12-21

**Authors:** Eri Muso, Tomomi Endo, Mitsuyo Itabashi, Hiroko Kakita, Yukako Iwasaki, Yu Tateishi, Toshiyuki Komiya, Toshiko Ihara, Wako Yumura, Takao Sugiyama, Kensuke Joh, Kazuo Suzuki

**Affiliations:** 1 Division of Nephrology and Dialysis, Kitano Hospital, The Tazuke Kofukai Medical Research Institute, 2-4-20 Ohgimachi, Kita-ku, Osaka, 530-8480 Japan; 2 4th Department of Internal Medicine, Tokyo Women Medical College, Tokyo, Japan; 3 Preventive Medical Center, Department of Nephrology, International University of Health and Welfare Hospital, Tochigi, Japan; 4 Division of Internal Medicine, Shimoshizu National Hospital, Chiba, Japan; 5 Division of Pathology, Sendai Shakaihoken Hospital, Miyagi, Japan; 6 Safety Control Department, University Hospital, School of Medicine/General, Medical Education Center, Teikyo University, Tokyo, Japan

**Keywords:** Anti-neutrophil cytoplasmic antibody, Vasculitis, Renal histology, Glomerulonephritis, Classification, Microscopic polyangiitis, Japan

## Abstract

The prognostic value of renal biopsy in anti-neutrophil cytoplasmic antibody (ANCA)-associated glomerulonephritis is widely recognized; however, there is no consensus regarding its pathological classification. Berden et al. proposed a new classification of glomerulonephritis in ANCA-associated vasculitis (AAV) categorized into focal, crescentic, mixed, and sclerotic classes and showed its prognostic value in 100 international multicenter cohorts for 1- and 5-year renal outcomes. In order to evaluate whether this new classification has predictive value and reproducibility in Japanese AAV cases, 87 cohorts with only microscopic polyangiitis in 3 limited centers in Japan were analyzed. In addition, those from Japan, Europe (Berden’s cohorts) and China were compared in a recent report.

## Introduction

We recently proposed pathological parameters of renal lesions observed in anti-neutrophil cytoplasmic antibody (ANCA)-associated vasculitis (AAV) patients [1]. The purpose of this proposal was (1) standardization of pathological findings in AAV should be authorized in Japan; (2) comparison with the European Vasculitis Study Group (EUVAS) standardization should be available; and (3) pathological parameters correlated with specific clinical findings should be evaluated (Table 1). As a result, the pathological parameters selected were almost compatible with those selected by EUVAS except for the collapse of glomeruli as the chronicity parameter; however, further evaluation using these parameters to investigate potential markers for the probability of end-stage renal disease (ESRD) is needed.

**Table 1:** Pathological parameters nominated for evaluation of active and chronic lesion in ANCA-related vasculitis in Japan (comparable with EUVAS)

*Glomerular lesion*	
No. of normal glomeruli	
Active lesion	Chronicity lesion
Mesangial proliferation	Sclerotic lesion
Endocapillary hypercellularity	Global sclerosis
Tuft necrosis	Segmental sclerosis
Cellular, fibrocellular crescent formation	Fibrous crescent
<50 %	<50 %
>50 %	>50 %
Rupture of Bowman’s capsule	Adhesion
	Collapse^a^
*Tubulointerstitial lesion*	
Active lesion	Chronicity lesion
Tubulitis	Atrophic tubule
Disruption of tubular basement membrane	Interstitial fibrosis
Interstitial cell infiltration	
Granulomatous lesion	
Peritubular capillaritis^a^	
*Vascular lesions*	
Active lesion	Chronicity lesion
Necrotizing	Arteriosclerosis
Endoarteritis	
Cell infiltration	
Thromboembolism	
Granulomatous lesion	

^a^Parameter not nominated in EUVAS

Among the parameters listed above, the number of normal or sclerotic glomeruli was proved substantially to be a prognostic indicator of renal outcome in accordance with basal renal function [2–4]; however, no sufficient consensus exists regarding the pathological classification. Recently, using some of the glomerular parameters, an international working group of renal pathologists proposed a new histopathological classification of glomerulonephritis (GN) in AAV with four categories (focal, crescentic, mixed and sclerotic), corresponding to the severity of renal function loss in this order during a 5-year follow-up [5]. As the evaluation was performed in 100 cases, consisting of 39 cases of granulomatosis with polyangiitis (GPA) and 61 cases of microscopic polyangiitis (MPA) in 32 centers in 9 European counties, the influence of the relatively mixed races and disease types could not be excluded. In Japan, >90 % of ANCA-positive GN is diagnosed as MPA, in which renal involvement is more frequent than in GPA, as previously reported [6]. In this study, we evaluated the predictive potential of this newly proposed categorization in myeloperoxidase (MPO)-ANCA-dominant MPA patients in Japan.

## Patients and methods

Eighty-seven patients with primary systemic vasculitis, in accordance with the Chapel Hill consensus criteria [7], diagnosed and treated from 2001 to 2010 in three centers (Kitano Hospital in Osaka, Tokyo Women Medical College in Tokyo and Shimoshizu National Hospital in Chiba) were analyzed. In all cases, renal biopsy was performed before treatment. Specimens including a minimum of 10 whole glomeruli were enrolled. Hematoxylin and eosin, methenamine silver, periodic acid-Schiff, and Masson trichrome staining were used for evaluation. The histological categorization based on glomerular lesion was performed following Berden’s group [5]—focal ≥50 % normal glomeruli, crescent ≥50 % of glomeruli with cellular crescents, sclerotic ≥50 % of glomeruli with global sclerosis, and mixed <50 % normal, <50 % crescentic, <50 % globally sclerotic glomeruli. A minimum of 6 months prognosis was observed for all patients. Renal and life survivals were analyzed at onset, 6 months, 1 year and 5 years after renal biopsy in available patients (87 at onset and 6 months, 84 at 1 year, 78 at 5 years).

## Results

### Patient profile and outcome in Japanese cohort

Median age was almost identical to the European study; however, males were dominant in Japan in contrast to a slight female dominance in Europe (Table 2).

**Table 2:** Comparison among evaluations of GN histological categories with clinical background in Europe, China and Japan

	European [5]	Japan	China [8]
Patients (number)	100	87	121
Centers (number)	32	3	1
Median age (range)	62.6 (20–80)	63.0 (17–85)	57.2 (15–81)
Male to female (number)	54:46	37:50	64:57
Clinical diagnosis (%)			
GPA	39 (39)	0	49 (40.5)
MPA	61 (61)	87 (100)	68 (56.2)
Renal-limited vasculitis	0	0	4 (3.3)
ANCA test (indirect immunofluorescence or ELISA)			
PR3-ANCA	45	0	13
MPO-ANCA	47	76	108
ANCA(−)	2	0	0
Missing	3	11	0
Median number of glomeruli per biopsy (range)	14.8 (10–49)	26.5 (10–98)	25.7 (NS)
Pathological classification number (%)			
Focal	16 (16)	40 (46.0)	33 (27.3)
Crescentic	55 (55)	7 (8.0)	53 (43.8)
Mixed	16 (16)	26 (29.9)	24 (19.8)
Sclerotic	13 (13)	14 (16.1)	11 (9.1)
Serum creatinine (mg/dl)			
Focal	NS	1.51 ± 1.49	2.22 ± 1.90
Crescentic		2.42 ± 1.67	5.01 ± 2.73
Mixed		3.37 ± 3.17	3.86 ± 2.69
Sclerotic		7.52 ± 4.92	8.51 ± 3.42
Death at 1-year follow-up	25/100	11/84	NS
Renal survival at 1-year follow-up			
Focal, crescentic, mixed, sclerotic (%)	93, 84, 69, 50	100, 86, 96, 35	100, 73, 83, 29
Renal survival at 5-year follow-up			
Focal, crescentic, mixed, sclerotic (%)	93, 76, 61, 50	100, 86, 96, 29	NS

Data of three patients were lost due to transfer to different hospitals before 1-year follow-up

*NS* not shown in the report

All cases in Japan had MPA; MPO-ANCA was positive in 76/87 (87.3 %). The median glomerular number was 26.5 in Japanese samples. At 6 months follow-up, 11 patients reached ESRD and a further 8 patients had died. At 1-year follow-up, no more patients had reached ESRD and a total of 11 patients had died. At 5-year follow-up, 18 patients had died and another 12 patients had reached ESRD.

### Classification of the renal biopsy in Japanese cohorts

**Fig. 1:**
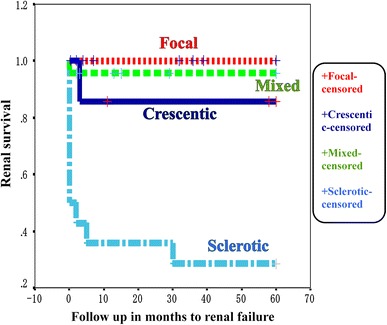
Renal survival (no development of end-stage renal failure) according to the four histologic categories in Japanese cohorts

In Japanese patients, almost half of the cases were categorized as focal (40/87; 46.0 %) with 14/87 (16.1 %) as sclerotic. Of the other 32 cases, only 7 (8.0 %) were categorized as crescentic, with the remaining 26 cases (29.9 %) being classed as mixed. As shown in Fig. 1, the Kaplan−Meier curve at the 5-year follow-up showed no increase of probability to ESRD in focal cases and a low increase in mixed cases; however, this increased with the ascending categories of crescentic and sclerotic GN.

### Comparison among evaluations of GN histological categories in Europe, China and Japan

The predictive value and reproducibility of this new classification from Japan, Europe and China were compared in a recent report [8]. As shown in Table 2, among the 100 respective patients (32 centers; Europe), 121 (1; China) and 87 (3; Japan), the GPA:MPA ratio was similar between Europe and China (39:61 and 49:64) in contrast to all MPA (0:87) in Japan. On the other hand, for serum ANCA positivity, MPO-ANCA positivity was dominant in China (89.1 %) and Japan (87.4 %) compared to Europe (45 %), where there was relatively high PR3-ANCA positivity (47 %) compared with China and Japan (10.7 and 0 %, respectively). The average numbers of glomeruli per case were significantly higher both in Japan (26.5) and China (25.7) than in Europe (14.8). The distribution of the four histological categories of GN were similar in Europe and China with crescentic cases being dominant (55 and 47 %, respectively), whereas in Japan, the number in this category was significantly lower (8.0 %). The probability of developing ESRD increased with the ascending categories of focal, crescentic, mixed, and sclerotic in Europe, and focal, mixed, crescentic and sclerotic in China. In Japan, as mentioned above, there was no increase of probability to ESRD in focal and mixed, but there was a high increased in sclerotic, as in Europe and China.

## Discussion

The histopathological findings of AAV in the kidney are considered to show a variety of lesions, of which crescentic and/or focal necrotizing GN as well as small-vessel arteritis are the most prominent [7]. In addition to the baseline laboratory data concerning renal lesions such as hematuria, proteinuria and decreased estimated glomerular filtration rate with systemic inflammatory signs such as C-reactive protein and organ involvement symptoms such as hemoptysis, renal histological findings have been expected to give highly reliable information not only to select the treatment protocol but to predict the outcome at baseline. Trials for the global standardization of active and chronic pathological parameters specifically in AAV have been performed not only in EUVAS but also in Japan, where a higher prevalence of MPA than EUVAS has been recognized, although the AAV prevalence itself is almost the same [9]. As shown in Table 1, these parameters are common findings in AAV. Almost all parameters are common in EUVAS selection, so our Japanese standardization of clinicopathologically critical parameters in AAV seems to be globally fulfilled.

The new classification of GN into four categories (focal, crescentic, mixed, sclerotic) by selecting some of the parameters of Berden et al. [5] was highly predictive in AAV patients from multicenters in Europe. In Japan, the significantly lower frequency of crescentic and relatively higher frequency of focal cases were noted; this might be partly attributed to the earlier intervention of renal biopsy after discovering a urinary or renal function abnormality in Japan. The relatively low creatinine level of the focal group in Japan compared with that of the same group in China might support this tendency. As the progression of renal injury tends to be different between MPA and GPA, comparisons should be performed only between MPA in Europe and in Japan. This was not possible in this classification study because there were no data on the ratio of MPA in the crescentic group in Europe. In this study, the Kaplan–Meier curve revealed the highly favorable prognosis of the mixed group. This indicates that the prognosis of this group is attributed to additional pathological parameter such as tubulointerstitial or vascular lesions nominated previously in Europe and Japan. At present, at least for MPA-oriented cohorts in Japan, this classification only by glomerular parameters might be insufficient to predict the probability of progressing to ESRD.

The comparison of European, Japanese and Chinese cohorts would be highly informative. The similarity of the GPA/MPA ratio between Europe and China in contrast to that of MPO-ANCA dominancy between Japan and China indicates that many GPA are MPO-ANCA-positive in China, as Chinese authors have stated. The GPA dominancy might be attributed partly to the localization of the center at a high latitude, which has been reported to be related to the high prevalence of GPA [10]. Although the numbers in the four categories were similar between Europe and China, there was a difference in the order of the increase of probability of progressing to ESRD between mixed and crescentic. The significantly more favorable prognosis of mixed than crescentic in China is similar to Japan, where both focal and mixed rarely showed progress to ESRD.

In conclusion, the mixed group in the new classification has high heterogenicity of histological activity and chronicity, which shows the insufficiency of this classification for prediction of the probability of progressing to ESRD. Re-evaluation of the predictive value by adding other parameters such as interstitial or vascular lesions for MPA-oriented cohorts is expected.
